# Structural and optoelectronic properties of NiOx thin films synthesized via co-precipitation for hole transport layer applications

**DOI:** 10.1038/s41598-025-18509-6

**Published:** 2025-10-06

**Authors:** Hairul Mardiah Hamzah, Norhayati Binti Soin, Yasmin Abdul Wahab, Md. Jakir Hossen, Manzoore Elahi M. Soudagar, Mohammad Nur-E-Alam, Mohammad Aminul Islam, Chenyoushi Xu, Mahaboob Patel

**Affiliations:** 1 Department of Electrical Engineering, Faculty of Engineering, Universiti Malaya, Jalan Universiti, 50603 Kuala Lumpur, Malaysia; 2 Nanotechnology Catalysis Research Centre, Universiti Malaya, Jalan Universiti, 50603 Kuala Lumpur, Malaysia; 3 Department of Physics, Hajee Mohammad Danesh Science and Technology University, Dinajpur, 5200 Bangladesh; 4 College of Engineering, Lishui University, Lishui, 323000 Zhejiang China; 5 Centre for Research Impact and Outcome, Chitkara University Institute of Engineering and Technology, Chitkara University, Rajpura, 140401 Punjab India; 6 Division of Research and Development, Lovely Professional University, Phagwara, Punjab 144411 India; 7 Institute of Sustainable Energy, Universiti Tenaga Nasional, Jalan Ikram-UNITEN, 43000 Kajang, Selangor Malaysia; 8 Centre for Promotion of Research, Graphic Era (Deemed to be University), Clement Town, Dehradun, India; 9 Miyan Research Institute, International University of Business Agriculture and Technology (IUBAT), Dhaka, 1230 Bangladesh; 10 Department of Mechanical Engineering, Wolaita Sodo University, 4620 Wolaita Sodo, Ethiopia

**Keywords:** PSCs, NiOx, Co-precipitation, Spin coating, HTL, Energy science and technology, Materials science, Nanoscience and technology, Physics

## Abstract

Perovskite solar cells (PSCs) have emerged as promising next-generation photovoltaic devices due to their high power conversion efficiencies and low fabrication costs. However, the performance and stability of PSCs are strongly influenced by the quality of charge transport layers, particularly the hole transport layer (HTL). This study investigates the structural, morphological, and optoelectronic properties of nickel oxide (NiOx) thin films prepared via a chemical co-precipitation method and applied as hole transport layers (HTLs) in perovskite solar cells. NiOx films were spin-coated and thermally treated at different calcination temperatures to evaluate their effect on phase formation, surface morphology, and interfacial compatibility. X-ray diffraction (XRD) confirmed the formation of cubic NiO with increased crystallinity at higher calcination temperatures, while FTIR spectroscopy revealed the transformation of Ni(OH)₂ to NiOx through the disappearance of hydroxyl bands and the appearance of metal-oxygen stretching vibrations. Surface morphology assessed by FESEM and morphology analysis by ImageJ showed that films calcined at 300 °C presented uniform and fine-grain structure, while the 400 °C samples exhibited coarsening and increased roughness. UV-Vis spectroscopy demonstrated variations in optical absorption and band gap narrowing with increasing crystallinity. These optoelectronic improvements are critical for efficient hole extraction and transport. The optimized film at 300 °C provided a balance between crystallinity, morphology, and surface quality, making it a promising candidate for enhancing the stability and efficiency of perovskite solar cells.

## Introduction

The rapid advancement of perovskite solar cell (PSC) technology has positioned this photovoltaic platform at the forefront of renewable energy research. With certified power conversion efficiencies (PCEs) now exceeding 25% (NREL, 2023), PSCs are regarded as strong candidates for next-generation solar conversion. Their exceptional optoelectronic properties of perovskite materials, including high absorption coefficients, tuneable bandgap, and long carrier diffusion lengths, have enabled unprecedented improvements in device performance^1^. However, the stability and efficiency of PCSs remain constrained by the properties of their charge transport layers, which are crucial for effective carrier extraction and transport within the device architectures^2^.

Nickel oxide (NiOx), a wide bandgap (~ 3.6–4.0 eV) p-type semiconductor with high hole mobility and excellent chemical stability, has emerged as a robust alternative to conventional organic hole transport layers such as Spiro-OMeTAD. Unlike organic HTLs, which suffer from moisture and thermal instability, NiOx offers long-term durability and compatibility with scalable, solution-based processing techniques such as spin coating and co-precipitation^3,4^. Nanostructured NiOx further enhances electrical conductivity and hole transport efficiency compared to its bulk form, thereby improving charge extraction from the perovskite absorber layer^5^.

The performance of NiOx HTL is highly dependent on their crystallinity, morphological and interfacial compatibility with the perovskite layer. Calcination temperature during synthesis is a critical parameter influencing particle size, phase purity, and electronic properties. While higher calcination temperature typically improve crystallinity and reduce defect density thereby enhancing hole mobility and reducing recombination losses. In addition, excessive heating can cause particle agglomeration, increased roughness, and non-uniform film coverage, all of which deteriorate interfacial quality and device performance^6–8^. Achieving an optimal thermal treatment is therefore essential for balancing crystallinity with smooth interfacial morphology to maximize PSC efficiency^9–12^.

Despite extensive research, there is no consensus on the optimal calcination temperature for NiOx that simultaneously provides high crystallinity and excellent interfacial properties. Furthermore, the correlation between NiOx structural quality and its photovoltaic performance remains insufficiently understood. To address these gap, this study systematically investigating the influence of calcination temperature on the structural, morphological, and optoelectronic properties of NiOx thin films synthesized via a surfactant-free, water-based co-precipitation method. This environmentally friendly approach avoids the use of organic additives while producing high-quality NiOx films suitable for large-scale manufacturing. The findings presented here provide valuable insights into processing-property relationships, offering a pathway for optimizing NiOx HTLs to improve the efficiency and stability or perovskite solar cells.

## Experimental methods

### Synthesis of NiOx nanoparticles

First, NiOx nanoparticles were prepared using the co-precipitation method. To synthesize NiOx, high-purity compounds like Ni(NO_3_)_2_·6H_2_O and NaOH were employed as precursors without additional purification. Ni(NO_3_)_2_·6H_2_O (0.5 M) were dissolved in deionized water and an aqueous NaOH (1 M) solution was added dropwise until pH ~ 10. The mixture then stirred for 1 h at room temperature. Nickel hydroxide (Ni(OH)₂) was formed in situ as a precursor via a chemical co-precipitation method by reacting aqueous nickel nitrate with a basic solution. The resulting Ni(OH)₂ precipitate was collected, washed, and then calcined at controlled temperatures to yield NiOx nanoparticles. The Green precipitate formed gradually and which was repeatedly rinsed with double distilled water and ethanol. The Green precipitate then left to dry at 80 °C overnight. After that, the developed NiOx were calcinated at 200 °C, 250 °C, 300 °C, 350 °C and 400 °C for 2 h. Finally, black NiOx powder was collected after calcination and used to prepare an aqueous NiOx ink for spin coating. The NiOx powder (1.5 wt%) was dispersed in deionized water and subjected to ultrasonication for 30 min to achieve uniform dispersion. The resulting colloidal suspension was passed through a 0.45 μm nylon syringe filter to remove any agglomerates. A few drops of the filtered ink were then deposited onto pre-cleaned FTO glass substrates and spin-coated at 3000 rpm. This procedure was adapted from a previously reported method^13^, with modifications introduced to improve dispersion quality and film uniformity.

### Characterization

Thermal analysis for NiOH to NiOx was analysed using TA instrument Q500 series from 40 °C to 500 °C at 5 °C/min heating rate under a nitrogen atmosphere. Bruker Alpha II FT-IR spectrometer with diamond ATR was used to analyze molecular compounds to identify the functional groups of NiOx. The absorbance spectra were analysed in the range from 500 nm to 4000 nm. PANalytical diffractometer with Cu Kα radiation target at 80 kV ranging from 10° to 80° was used to determine the crystallinity of NiOx. The morphology and elemental analysis of NiOx were distinguished by FESEM-EDX Zeiss Gemini Auriga. The optical and electrical properties of NiOx as HTL were characterized using a PerkinElmer LAMBDA 1050 UV-Vis spectrophotometer and the Ecopia Model HMS-3000 for Hall effect respectively.

## Results and discussion

### Structural evolution of NiOx with calcination temperature

#### TGA analysis

**Fig. 1:**
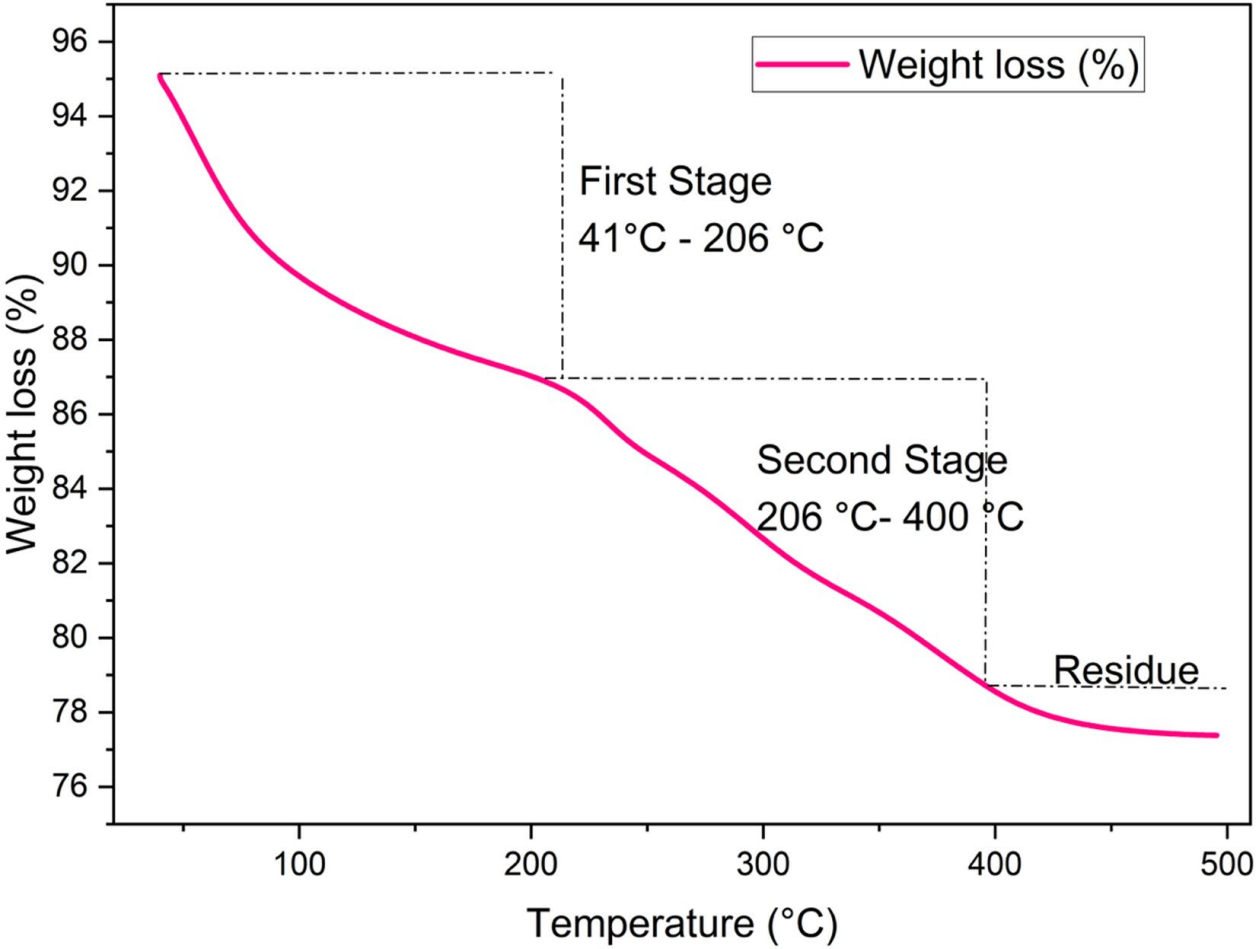
Thermal analysis of Ni(OH)_2_ for its conversion into NiOx.

The TGA curve shown above illustrates the thermal decomposition behaviour of nickel hydroxide (Ni(OH)_2_) during the conversion to nickel oxide (NiOx). The analysis was performed under a controlled heating program, with weight loss recorded as a function of temperature. From Fig. 1 presented below, a noticeable weight loss begins at 41 °C and continues until approximately 125 °C. This stage accounts for the evaporation of physically adsorbed moisture and surface-bound water on the Ni(OH)_2_ particles. The weight loss rate is relatively high in this region, indicated by a peak rate of 6.89%/min around 41 °C. This process known as a desorption process where no structural transformation occurs at this stage. At an intermediate weight loss occurs between 125 °C and 210 °C, a slower and more gradual weight loss is observed. This is due to the release of chemically bound water, indicating the onset of dihydroxylation of Ni(OH)_2_. Minor structural rearrangements may begin here, leading into the main decomposition phase. Major decomposition phase was observed between 200 °C and 400 °C where a significant and rapid weight loss occurs with a peak rate of 3.27%/min at around 226 °C. This stage corresponds to the thermal decomposition of Ni(OH)_2_ to NiOx as shown in Eq. (1) below:

1 $$\:Ni{\left(OH\right)}_{2}\to\:NiO+{H}_{2}{O}_{\left(g\right)}$$

This transformation is crucial for achieving phase for pure NiOx, as it removes hydroxyl groups and forms the desired oxide structure. After 400 °C, the curve shows minimal further weight loss, indicating that NiOx formation is complete. The material becomes thermally stable, and no further decomposition is detected up to 500 °C. Based on this TGA analysis, a calcination temperature round 300 °C is optimal to ensure complete transformation while maintaining the physical or chemical quality of the NiOx.

#### FTIR analysis

Although FTIR spectroscopy is not capable of determining exact oxidation states or electronic environments, it is a powerful technique for identifying functional groups and vibrational modes associated with specific chemical bonds. In this study, FTIR was used to confirm the presence and evolution of hydroxyl (–OH) groups and metal oxygen (Ni–O) bonding during the conversion of Ni(OH)₂ to NiOx. The appearance of Ni–O vibrational bands at ~ 470 cm⁻¹, along with the diminishing O–H stretching vibrations around 3400 cm⁻¹, provides qualitative evidence of the structural transformation. However, we acknowledge that XPS is the appropriate tool for determining elemental composition, oxidation states (e.g., Ni²⁺ vs. Ni³⁺), and surface chemical states, which are beyond the scope of this current FTIR-based analysis.

**Fig. 2:**
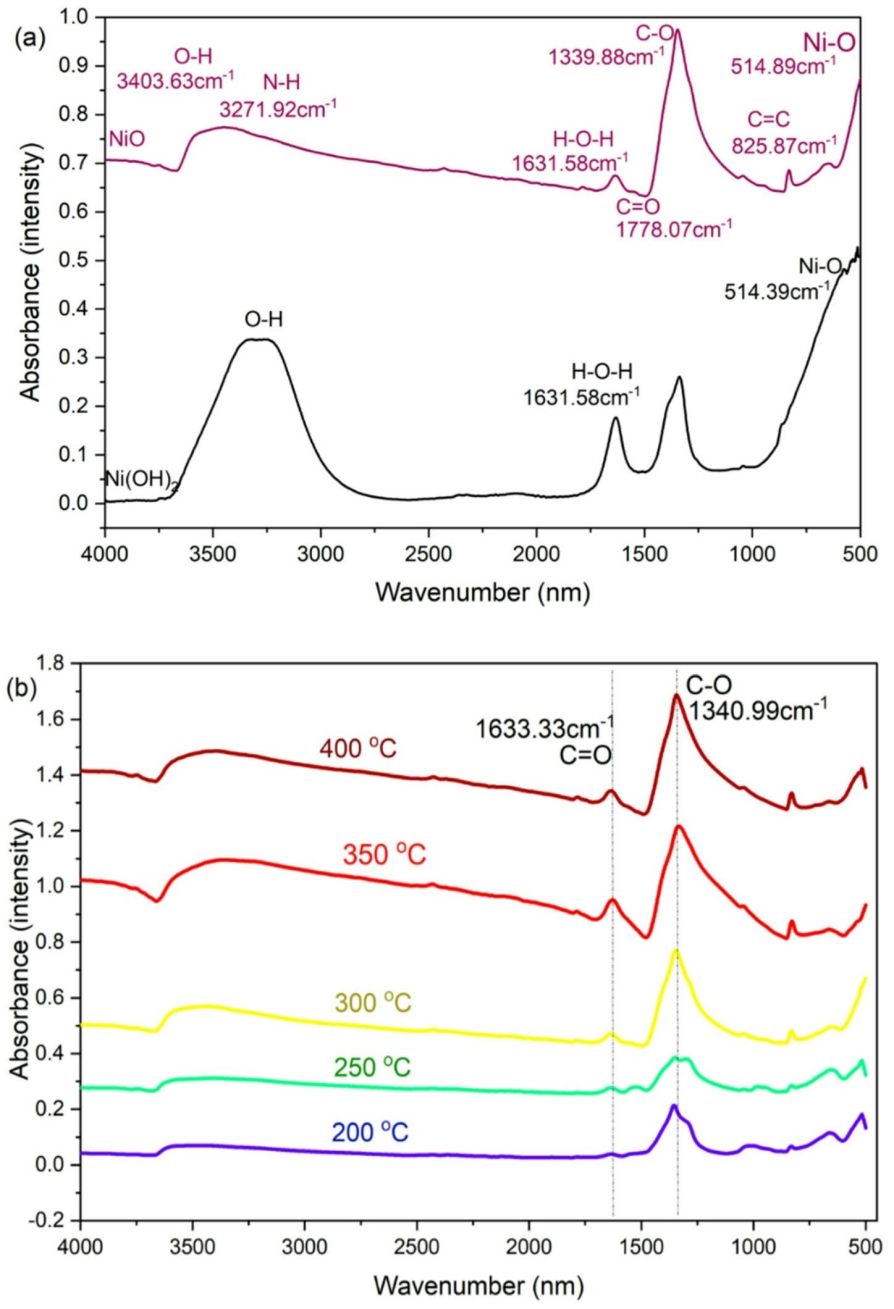
FTIR spectra of (a) formation of NiOx from Ni(OH)_2_ (b) different calcination temperatures of NiOx.

Ni(OH)_2_ and its calcinated product, referred to as NiOx (primarily NiOx formed through heat treatment), were analyzed using Fourier transform infrared (FTIR) spectroscopy to understand how their chemical bonds and functional groups change with temperatures. Figure 2(a) shows the FTIR spectra for both the before and after calcinated samples at various temperatures. The FTIR spectra confirmed the formation of NiOx from Ni(OH)_2_ by the co-precipitation method. Table 1 presents a list of obtained FTIR peak vibrations for both Ni(OH)_2_ and NiOx.

Ni(OH)_2_ shows significant peaks for water molecules at 3200 cm^−1^ and H-O-H bending vibration at 1631.58 cm^−1^. The water molecules peaks shift down after the calcination process at 300 °C. NiOx FTIR spectrum exhibits significant absorption peaks at 514.89 cm^−1^ for Ni-O stretching, 1339.88 cm^−1^ for C-O stretching, and 3271.92 cm^−1^ for N-H stretching. These vibrational peaks matched those reported in the literature by Asif Hayat et al. (2019), who synthesized NiOx using the microwave radiation method^14^.

Figure 2(b) presents the FTIR spectrum corresponding to various calcination temperatures. The absorption intensity for C-O and C = O peaks increases as the temperature rises from 200 °C to 400 °C. The width of the band suggests the nanocrystalline characteristics of the samples^7^. It should be noted that the broadening of IR absorption bands in the FTIR spectrum is not used to determine particle or crystallite size. Instead, the nanocrystalline nature of the NiOx films was confirmed by analysing the broadening of XRD peaks, which is a more direct and widely accepted method for estimating crystallite dimensions. Elevating the calcination temperature enhances the peaks for Ni-O vibrational bands in the FTIR of NiOx powder. At 200 °C and 250 °C, the NiOx was not fully formed yet as the water molecules were visible. The NiOx was fully formed at 300 °C as the peak for C-O is intense.

**Table 1:** FTIR bands spectrum identified in Ni(OH)2 and NiOx.

IR Vibrations	Wavenumber (cm^−1^)
Ni-O stretching	514.89
C = C stretching	825.87
C-O stretching	1339.88
H-O-H bending vibration	1631.58
C = O stretching	1778.07
N-H stretching	3271.92
O-H water molecules	3403.63

#### X-ray diffraction (XRD) analysis

**Fig. 3:**
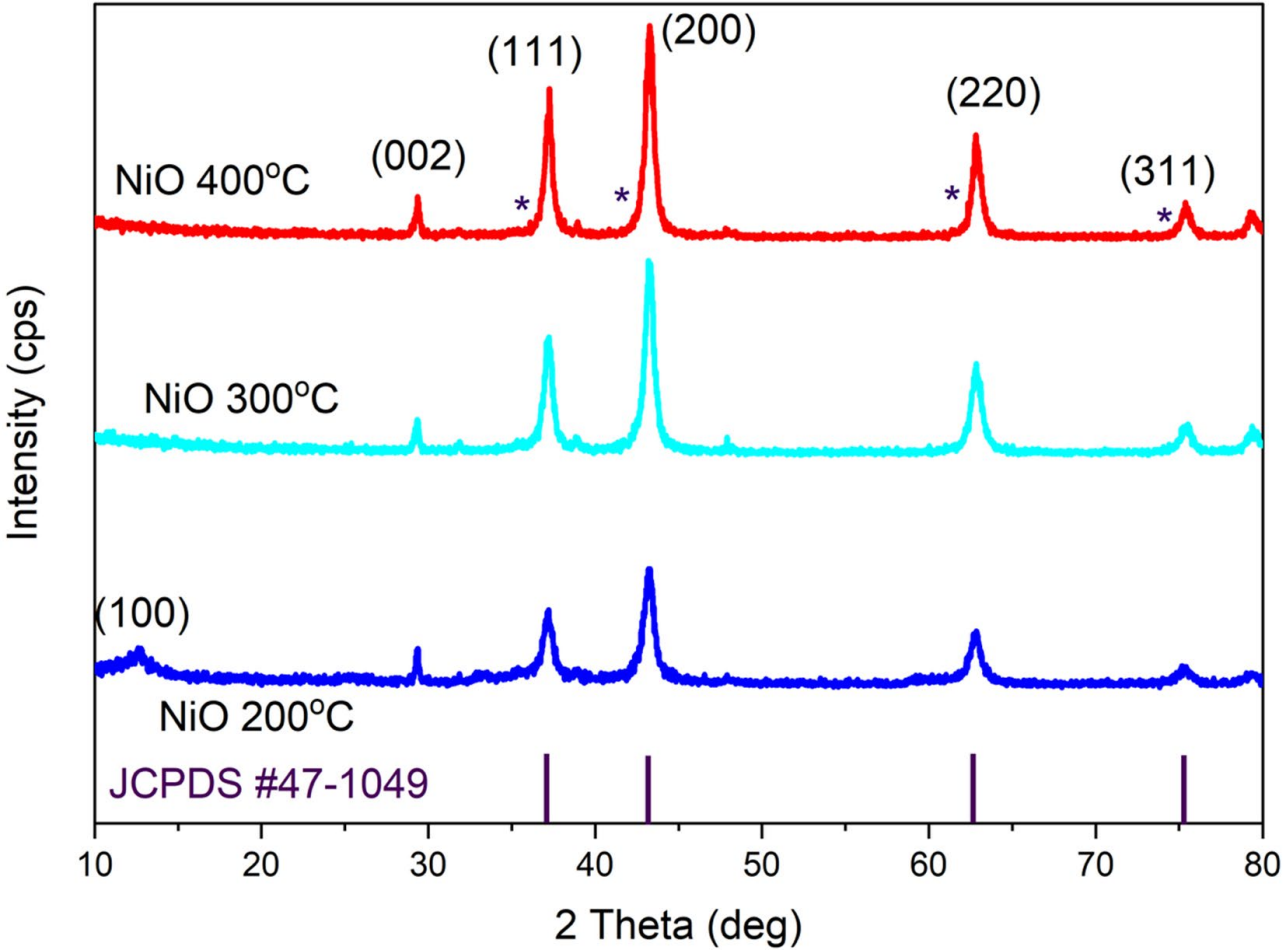
XRD pattern of NiOx thin films alongside reference diffraction peaks for cubic NiO (JCPDS No. 47-1049). The reference peaks are marked to verify phase purity.

X-ray diffraction was used to examine the crystallographic characteristics of NiOx which is a cubic crystal structure. Each Ni^2+^ ion forms a mixture of corner and edge-sharing NiO_6_ octahedral bonds with six O_2_^−^ions^15^. The XRD pattern in Fig. 3 reveals the presence of octahedral crystal structure followed by the reflection index to face-cantered cubic phase NiOx (JCPDScard no #47-1049). The (111), (200), (220), and (311) diffraction planes are represented by the three characteristic peaks at 37.16°, 43.2°, 62.8°, and 75.3° respectively. At 200 °C, the XRD pattern shows a weak (100) diffraction peak, suggesting that the NiOx sample possesses low crystallinity rather than being fully amorphous. This indicates it is in a transition phase, where some small, ordered domains a beginning to form, but the overall structure is still mostly disordered. Crystallization of NiOx became evident at 300 °C, as indicated by the appearance of sharp and distinct XRD peaks. The Debye-Scherrer method was used to calculate the crystallite size of the synthesized NiOx. The average crystallite size were determined using the full-width half maximum (FWHM) values, indicating the ratio of the root-mean-fourth-power to the root-mean-square value of the thickness. The average crystallite sizes were determined with the Debye Scherrer in Eq. (2) as follows:

2 $$\:\text{D}\text{S}=\frac{K}{Cos}$$

Where, λ represents the X-ray wavelength utilized (1.5406 Å), K denotes the shape factor, which is 0.9, θ indicates the angle of diffraction, and β is defined as the full width at half maximum (FWHM) of the corresponding XRD peak located at 2theta.

Table 2 tabulated the peak position, FWHM, crystallite size of each peak, and the average crystallite size for NiOx. From 200 °C to 300 °C the crystallite size was found to be reduced from 9.59 nm to 9.19 nm but the size increased back to 10.67 nm at 400 °C. Ni(OH)_2_ had a bigger crystallite size than NiOx and increasing the crystallinity of NiOx increased the particle size too^16^. Thus, the NiOx with calcination temperature at 300 °C had a smaller particle size thus producing higher surface areas.

X-ray diffraction (XRD) patterns of the NiOx samples calcined at different temperatures revealed the typical cubic NiO phase with characteristic diffraction peaks at 2θ = 37.7° and 61.6°, corresponding to the (111) and (220) planes, respectively. As the calcination temperature increased, the intensity and sharpness of these peaks improved, indicating enhanced crystallinity and growth of NiOx crystallites. The crystallite size calculated from the Scherrer equation showed a progressive increase with temperature, confirming the grain growth phenomenon.

However, for the sample annealed at 400 °C, although the NiOx phase was fully developed as supported by the completion of hydroxide decomposition observed in TGA the material exhibited signs of overgrowth. Excessive calcination at this temperature likely promoted crystal coarsening and possible particle agglomeration, leading to a reduction in surface area. This overgrowth may compromise the film’s morphology by causing surface roughness and poor perovskite wettability, which are critical for interface quality and effective hole extraction^17^. Hence, while the 400 °C sample demonstrates high crystallinity, it may not be optimal for photovoltaic performance due to these structural Limitations. An ideal balance between phase purity, particle size, and interfacial smoothness is therefore better achieved at moderate calcination temperatures such as 300 °C.

**Table 2:** The crystallite size of NiOx based on different calcination temperature. Used or crystallite size via scherrer equation.

200 °C peak position (2 theta)	FWHM	Crystallite size D (nm)	D nm (Average)
37.16	0.888	9.442	9.594
43.21	0.882	9.689	
62.79	0.955	9.750	
75.32	1.057	9.495	

300 °C peak position (2 theta)	FWHM	Crystallite size D (nm)	D nm (Average)
37.19	0.707	10.651	9.192
43.25	0.746	9.902	
62.86	0.840	8.065	
75.41	0.771	8.149	

400 °C peak position (2 theta)	FWHM	Crystallite size D (nm)	D nm (Average)
37.21	0.624	12.074	10.668
43.27	0.670	11.022	
62.88	0.689	9.840	
75.41	0.646	9.735	

**Fig. 4:**
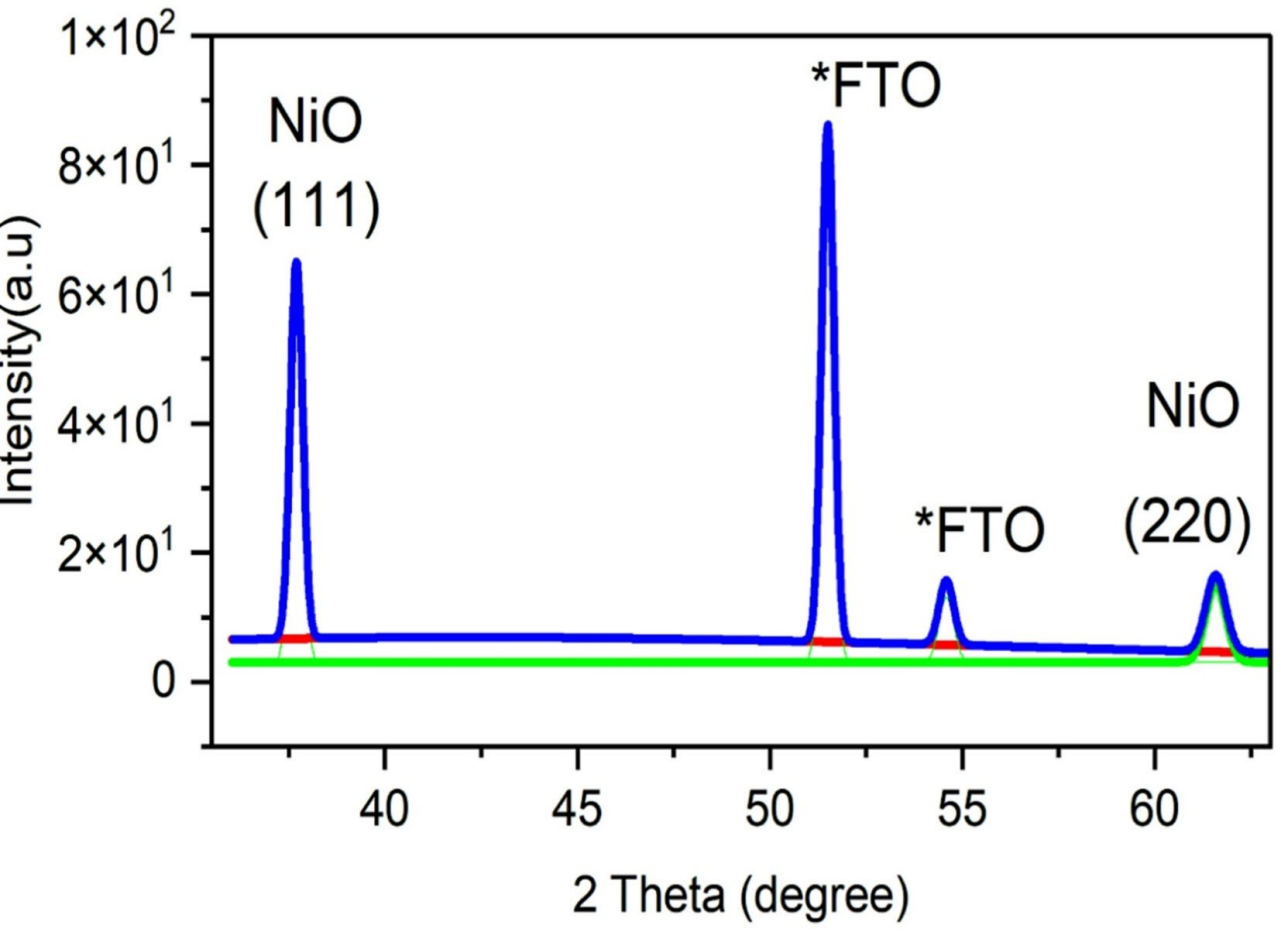
Deconvolution peaks for FTO/NiO.*correspond to SnO_2_ from the FTO substrate (AMCSD 0011761).

To isolate and identify the contribution of the NiOx film from the underlying FTO substrate, deconvolution of the XRD pattern was performed. This approach allowed us to resolve overlapping peaks and better assess the phase purity and crystallinity of the NiOx layer. Table 3 presents the deconvolution details obtained from XRD analysis of FTO/NiO films, showing how calcination temperature influences peak positions, FWHM values, and estimated crystallite sizes. Figure 4 illustrates the XRD deconvolution of NiOx dispersed on the FTO glass by the spin coating method. From the figure, the diffraction plane of (111) and (220) from NiOx was found at 37.70° and 61.58°, respectively. To verify the phase purity of the NiOx films and ensure that no residual hydroxide or intermediate oxide phases were present, the XRD patterns were compared with standard SnO₂ reflections from the FTO substrate. Reference peak positions were taken from the American Mineralogist Crystal Structure Database (AMCSD No. 0011761 entry matching rutile SnO₂), including prominent peaks at 2θ = 26.61°, 33.91°, and 37.99°. These were distinguishable from the NiOx (111) and (220) peaks at 2θ = 37.70° and 61.58°, confirming the successful formation of cubic NiOx without impurities.

**Table 3:** The Deconvolution details from XRD analysis of fto/nio.

FTO/NiO			
Peak index	2 Theta (degree)	Max height	FWHM
1	37.70	58.32	0.37
2	51.50	80.02	0.38
3	54.58	9.99	0.45
4	61.58	11.91	0.61

### Surface morphology

Field Emission scanning Electron Microscopy coupled with Energy-Dispersive X-ray Spectroscopy (FESEM-EDX) was employed to examine both the morphology and elemental composition of the NiOx samples. While EDX confirmed the presence of Ni and O, its limitations in accurately quantifying light elements such as oxygen preclude its use for precise stoichiometric determination. Therefore, EDX results were not used for stoichiometric determination. Instead, TGA was employed to monitor the thermal decomposition of Ni(OH)₂ to NiOx, providing more reliable insight into the oxygen-related weight loss and phase conversion process.

**Fig. 5:**
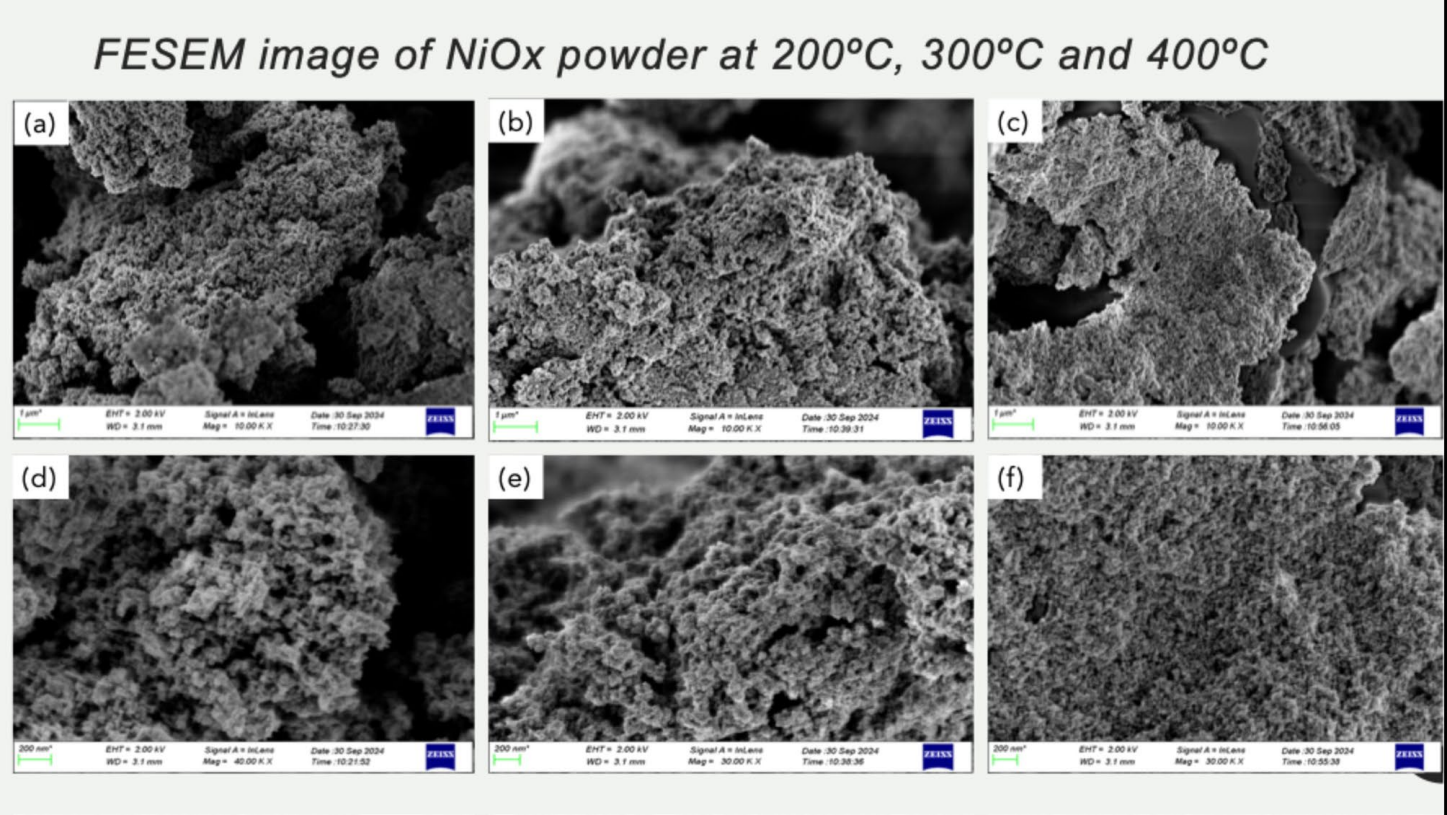
FESEM micrograph of NiOx at 200 °C, 300 °C, and 400 °C for (a-c) at 10 K magnification and (d-f) at 30 K magnification.

The surface morphology (as shown in Fig. 5) of as-prepared NiOx for calcination at 400 °C shows relatively coarse and less-defined grain compared to the more compact and uniform grain structure observed for 200 °C and 300 °C calcination temperatures. NiOx shows better grain structure. The grain size distribution was analyzed using ImageJ software for FESEM images, with at least 30 particles measured per sample across multiple image regions to account for spatial variation. The average grain size was found to be 1715.91 ± 85 nm for 200 °C, 3313.17 ± 112 nm for 300 °C, and 3726.35 ± 124 nm for 400 °C respectively. The reduction of lattice defects was particularly notable after calcination at 300 °C, resulting in improved particle arrangement, while the 400 °C sample showed more agglomeration despite its uniformity. These observations are consistent with the findings from Boukhachem et al. (2014), who reported that the surface roughness of NiOx decreases with the increasing temperature from 350 °C to 450 °C^18^.

**Table 4:** Energy dispersive X-ray analysis of NiOx prepared from different calcination temperatures.

Sample	Element	Wt %	At%
NiO 200 °C	Ni	25.03	55.05
	O	74.97	44.95
NiO 300 °C	Ni	18.4	45.28
	O	81.6	54.72
NiO 400 °C	Ni	24.47	54.32
	O	75.53	45.68

**Fig. 6:**
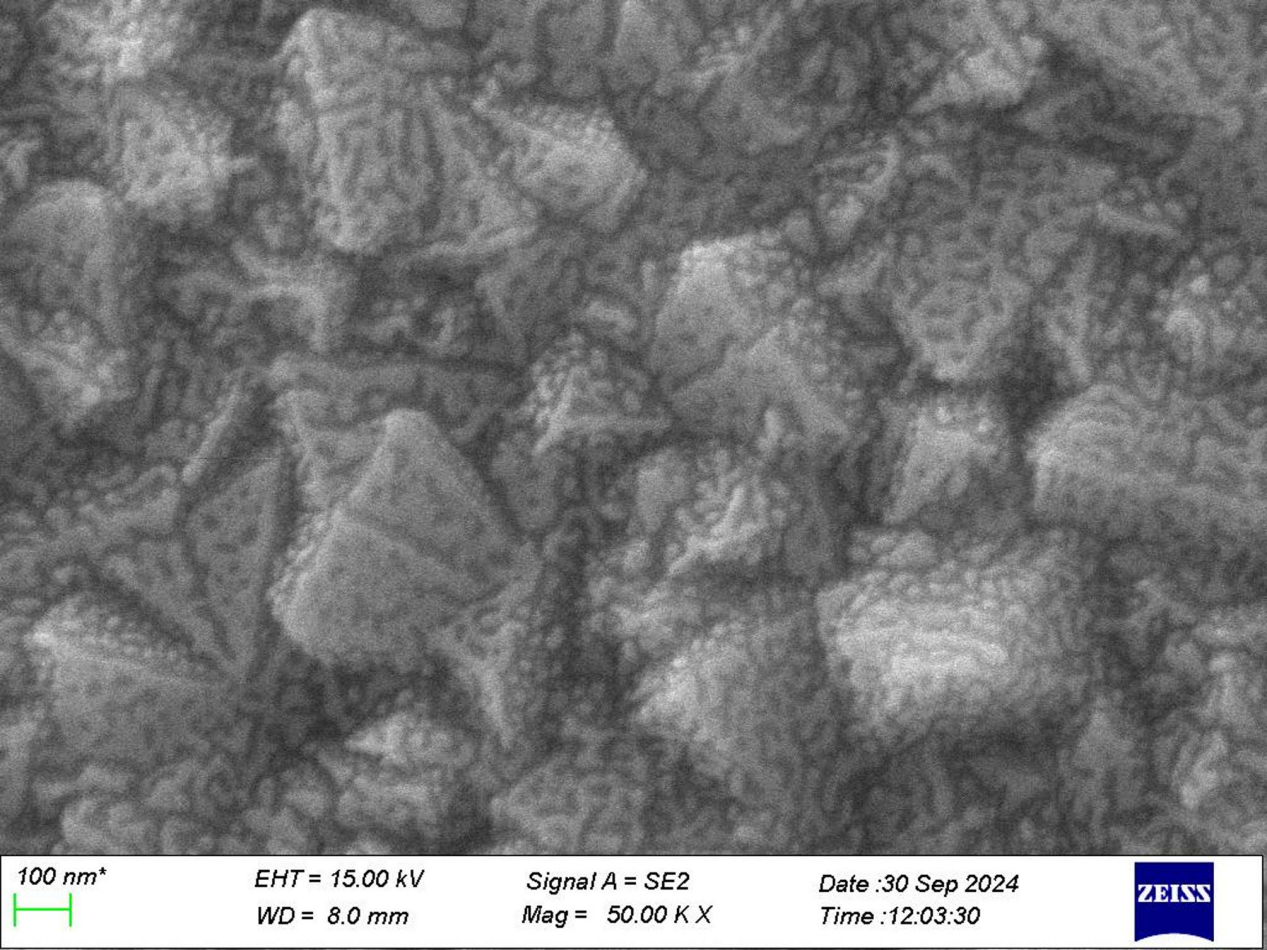
Top-view image of NiOx calcinated at 300 °C spin-coated on FTO glass.

The EDX analysis in Table 4 shows an elemental analysis of the prepared NiOx with different calcination temperatures. The EDX profiles indicate the percentages of nickel and oxygen, with no other elements present as impurities in the synthesis of NiOx. The oxygen element was the highest at 300 °C at 81.6 wt%, whereas for 200 °C and 400 °C, the oxygen element showed 74.97 wt% and 75.53 wt%, respectively. This concludes that NiOx prepared at 300 °C, the rock salt structure formed with O^2−^ ions bonded to Ni^2+^ ions at the edges and corners of the octahedral with a non-stoichiometric structure. Figure 6 below illustrates the top-view micrograph shows the FTO glass was coated with NiOx layer. The NiOx was well dispersed on the surface of the FTO glass.

The optimized NiOx-based PSCs in this work exhibit structural and optoelectronic characteristics in agreement with previous report, where NiOx HTLs have enabled device efficiencies exceeding 20%^19^. In contrast to organic HTLs such as Spiro-OmeTAD, which typically require hydroscopic dopants for example 4-tert-butylpyridine (tBP) or lithium bis(trifluoromethanesulfonyl)imide (Li-TFSI) that accelerate degradation under humid conditions^20,21^, NiOx demonstrates superior chemical stability and eliminate the need for such additives. When compare with other inorganic HTLs, including Cu_2_O and Co_2_O_4_, NiOx offers a wider bandgap (~ 3.6-4.0 eV), thereby minimizing parasitic absorption within the visible spectrum, while still enabling low-temperature, solution-based processing^22^. These combined attributes underscore NiOx as a robust, efficient, and scalable alternative for advanced PSC architectures.

### Optoelectronic properties

**Fig. 7:**
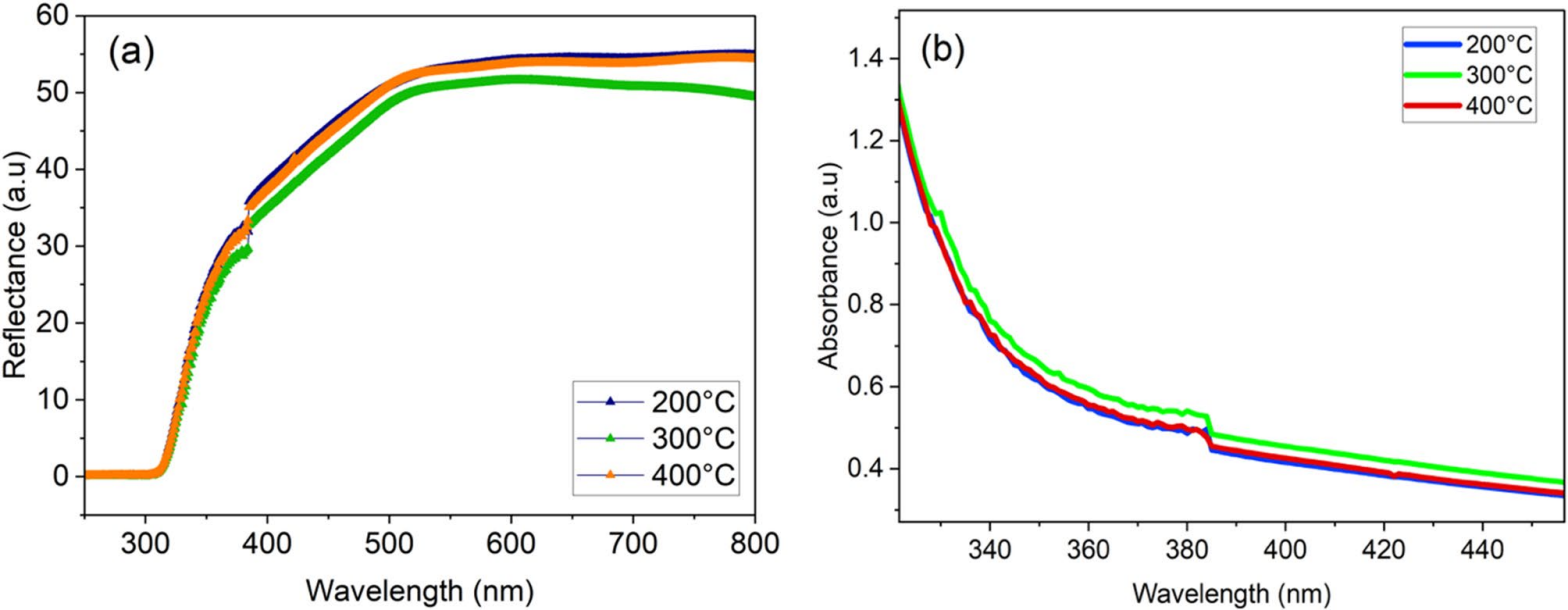
Reflectance (a) and absorbance (b) spectra of UV-VIS for NiOx prepared by chemical co-precipitation method at 200 °C, 300 °C, and 400 °C calcination temperature.

Ultraviolet-visible (UV-Vis) spectra were employed to analyze the optical properties of NiOx samples. UV-visible reflectance and absorbance spectroscopy are significant techniques utilized to examine the optical characteristics of semiconducting materials. The UV-vis reflectance and absorbance spectra for three NiOx samples were measured in the 250–800 nm range and illustrated in Fig. 7. The UV-Vis spectra show NiOx calcinated at 300 °C had slightly lower reflectance and higher absorbance of Light as compared to NiOx with 200 °C and 400 °C calcination temperatures. Although the absorbance spectra in Fig. 7 appear similar., this trend is consistent with previous studies reporting that moderate variations in calcination temperature (200–400 °C) cause only minor changes in the optical absorption edge of NiOx thin films due to their wide bandgap nature^23^. The slight shifts observed may be attributed to gradual improvements in crystallinity and reduction in defect states rather than drastic changes in the electronic band structure^24^. Earlier findings from Pranwadee et al.., 2018 indicated that NiOx with a smaller particle size and larger surface area exhibits strong UV-Vis absorption^25^. This concludes that NiOx prepared at 300 °C has better surface area after spin coating on the FTO to interact with particles of solar energy or photons, thus demonstrating better light absorption and less reflectance. In addition, the decrease in reflectance of the products can be attributed to the improvement of lattice defects, leading to increased absorption^26^.

In addition to structural and optical improvements, the electrical properties of the NiOx films are essential for evaluating their suitability as hole transport layers in PCSs. In this study, the improved optoelectronic behaviour observed through UV-Vis absorption and band gap narrowing suggests enhanced electronic performance, particularly in the sample calcinated at 300 °C. This enhancement can be attributed to increased crystallinity and reduced defect density, both of which support improved hole mobility. Literature supports that the electrical conductivity of NiOx increases with calcination temperature up to an optimal point, as higher thermal energy reduces structural disorder and promotes grain connectivity. For instance, Ming et al. reported that solution-processed NiOx films exhibited a significant increase in conductivity (from 10⁻⁷ to 10⁻⁴ S/cm) when annealed at temperatures between 250 and 350 °C, directly correlating to improved PCEs in PSCs^6^. Similarly, Jeon and Park (2025) observed optimal device performance when NiOx was calcinated at around 300 °C due to the balance between crystallinity and smooth surface morphology^27^.

In our work, the UV-Vis data for the 300 °C film also revealed slight bandgap narrowing (from ~ 3.68 eV to ~ 3.54 eV), which is consistent with the presence of sub-band states that facilitate hole transport. These observations imply that NiOx treated at 300 °C has more favorable electrical properties, aligning well with previous findings and confirming the effectiveness of the chemical co-precipitation synthesis followed by moderate thermal treatment. By contrast, the 400 °C sample likely suffers from reduced interfacial contact due to grain overgrowth and increased surface roughness, which may lead to non-uniform current distribution and recombination losses when integrated into device architectures.

#### Band gap analysis

**Fig. 8:**
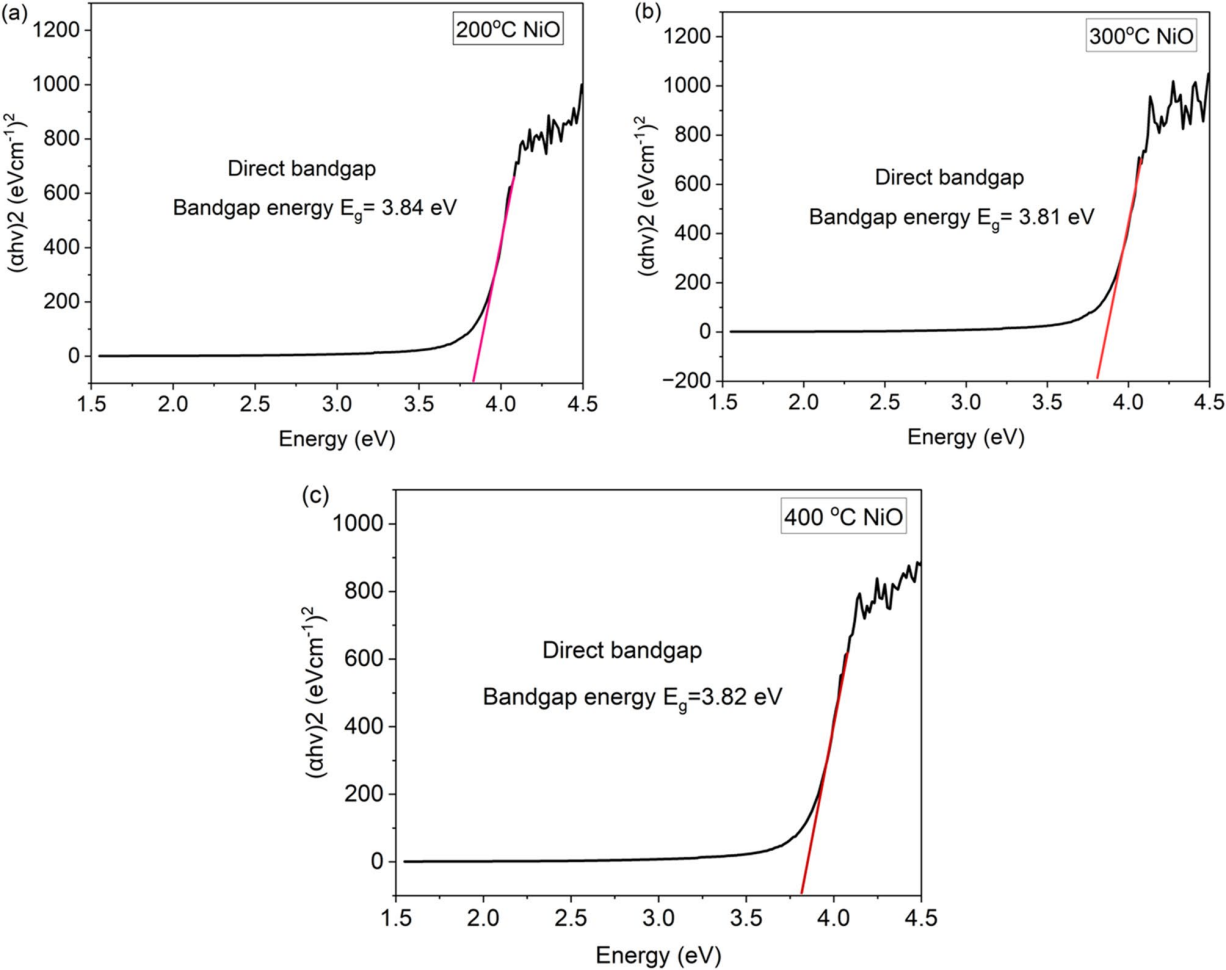
Tauc plot for NiOx films calcinated at (a) 200 °C, (b) 300 °C and (c) 400 °C. The band gap values were obtained by extrapolating the linear region of (αhv)^2^ vs. hv. Error bar indicate the standard deviation from three independent measurement.

The energy bandgap is depicted in Fig. 8. The energy bandgap values for three samples are ascertained using the Tauc relation in Eq. (3):

3 $$\:ahv={A(hv-{E}_{g})}^{n}$$

where ν denotes the frequency of the light source, α represents the absorption coefficient, A is a constant (independent of n), $\:h$ signifies Planck’s constant, and $\:n$ is the exponent determined by the quantum selection rules. The exponent $\:n$ takes the value of $\:\frac{1}{2}$ for direct allowed transitions and 2 for indirect allowed transitions. In the present study, an exponent of $\:n=\frac{1}{2}$ was employed, as nickel oxide (NiOx), particularly in its nanocrystalline and thin-film forms, is widely reported to exhibit a direct allowed bandgap. This assumption is consistent with previous literature, which demonstrates that NiOx prepared via chemical or low-temperature methods tends to display direct optical transitions. The Tauc relation is a technique for ascertaining the band gap of a substance using its absorption spectra.

The bandgap for NiOx is between 3.6 and 4.0 eV^28^. The direct bandgap calculated from the Tauc plot relation is 3.84, 3.81, and 3.82 eV for 200 °C, 300 °C, and 400 °C, respectively. The results show that the NiOx spin-coated on the FTO glass enables the electrons to transition from the valence band to the conduction band without any change in momentum due to direct bandgap materials having strong optical transitions. From the results, NiOx produced from 300 °C calcinated temperature has the lowest value and is suitable for the spin coating method. For a good semiconductor, a narrow bandgap is essential for high mobility of electrons to be transported to the absorber layer. Furthermore, the composition engineering and dimensional approaches present effective strategies for optimizing band gaps in alignment with the solar energy spectrum, thereby improving the performance and stability of perovskite solar cells (PSCs)^29^.

Although the Tauc plots in Fig. 8 indicate slight variations in the optical band gap with increasing calcination temperature, the extracted values are close to each other, with differences falling between the range of the experimental uncertainty. The relatively large error margins observed are mainly attributed to instrumental resolution, baseline noise, and the manual selection of the linear fitting range in the (αhv)^2^ versus hv plots. Similar small variations have been reported in literature for NiOx thin film calcined over moderate temperature ranges, as their wide bandgap is largely preserved unless significant changes in stoichiometry or phase occur^30,31^. To minimize uncertainty, multiple measurements were performed and averaged. However, due to the inherently small band gap shifts, the resolution of this method remains limited. Further improvement in precision could be achieved through higher resolution UV-Vis measurements and automated fitting algorithms, which may better resolve subtle optical transitions.

#### Urbach energy

Adjacent to the absorption coefficient curve and near the optical band edge, an exponential segment is referred to as the Urbach tail. The exponential tail is present in low-crystalline, poorly crystalline, disordered, and amorphous materials due to localized states extending into the band gap. It is an essential measurement in crystalline materials as it indicates the degree of order or disorder within the material. It pertains to the existence of localized states arising from the upper portion of the valence bands that extend into the band gap at low photon energy^32^.

The spectrum dependence of the absorption coefficient (α) on photon energy (hv) in the low photon energy range is referred to as the Urbach empirical rule, represented by the Eq. (4):

4 $$\:a=ao\:exp\:\left(\frac{hv}{EU}\right)$$

where αo is a constant and EU represents the energy of the band tail, often referred to as Urbach energy. This parameter exhibits a weak temperature dependence and is frequently interpreted as the width of the band tail resulting from localized states within the typical band gap associated with disordered or poorly crystalline materials. By applying the logarithm to both sides of the final equation, one can get a linear equation. It is presented in Eq. (5) as follows:

5 $$\:\text{ln}a=\text{ln}{a}_{0}+\left(\frac{hv}{EU}\right)$$

**Fig. 9:**
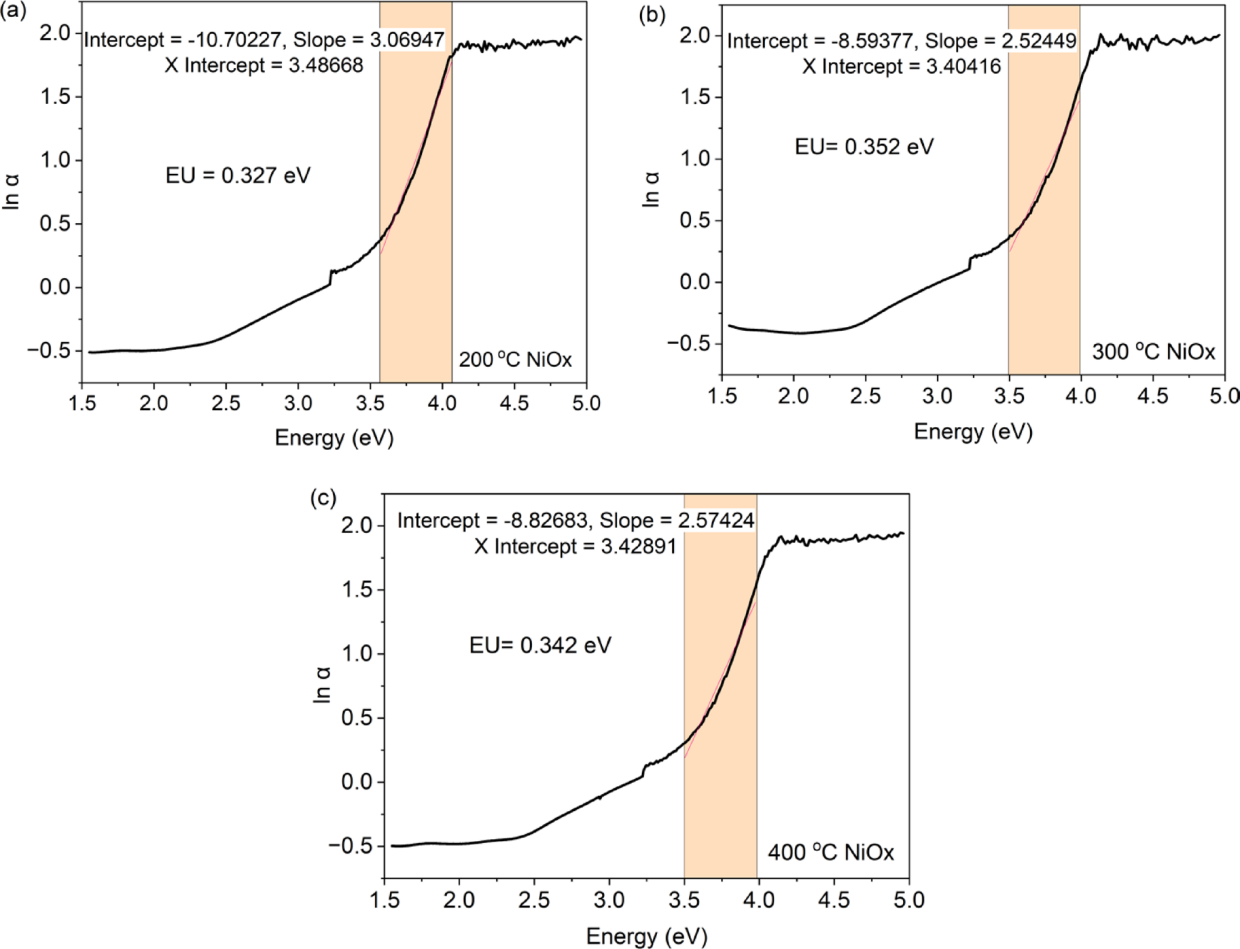
Urbach energy analysis for NiOx calcinated at (a) 200 °C, (b) 300 °C and (c) 400 °C.

Consequently, the band tail energy, or Urbach energy (EU), can be derived from the slope of the linear plot of ln(α) vs. the incident photon energy (hν). Figure 9 shows the Urbach energy determined from the Urbach empirical rule. The Urbach energy for NiOx calcinated from 200 °C to 300 °C increased from 0.327 eV to 0.352 eV, but decreased to 0.342 eV as the calcination temperature increase to 400 °C for the spin coating technique. This trend agree to the relationship of band gap energy and Urbach energy, which is an inverse relationship. The lower band gap will have a wider band tail. The findings conclude that at 300 °C of NiOx calcination temperature, the NiOx has a slightly higher structural disorder, and the optimum temperature for NiOx spin-coated on the FTO glass.

It should be noted that the spectral range selected for sample at 200 °C differs slightly from those used for 300 °C and 400 °C samples. This adjustment was made to exclude regions affected by spectral noise, ensuring a reliable and stable linear fitting of the absorbance edge for that specific dataset. Although identical range would facilitate a more direct numerical comparison, the present approach prioritizes the accuracy of individual fittings. The small variations observed in Urbach energy values among the samples are within the range reported for NiOx thin films in similar studies^33^. This observation indicates that moderate calcination temperature changes do not drastically affect the density of localized states. Therefore, while the numerical differences are minimal, the results still reflect the general trend of slightly reduced disorder with increasing calcination temperature^34^.

#### Hall effect study

The electrical conductivity σ of the NiOx films is governed by the relation in Eq. (6) below:

$$\:\sigma\:=qn\mu\:$$

**Fig. 10:**
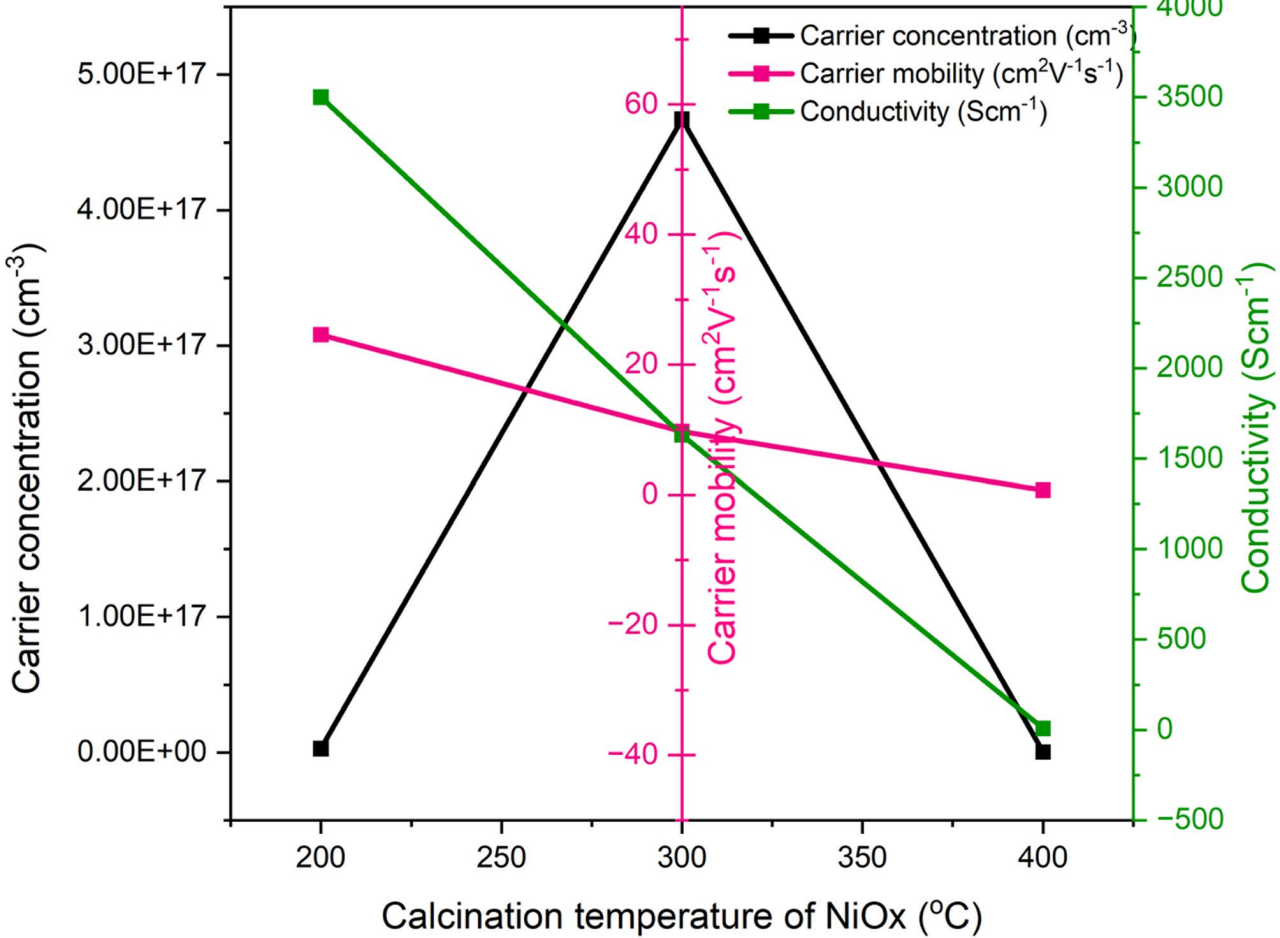
Hall effect of NiOx prepared at different calcination temperatures coated on FTO glass.

Where q is the elementary charge, $\:n$ is the carrier concentration, and $\:\mu\:$ is the carrier mobility. The hall measurements in Fig. 10 indicate a non-monotonic dependence of these parameters on calcination temperature. The carrier concentration reaches a maximum at 300 °C, while mobility and conductivity show different trends with temperature. Several physical mechanisms can observe defect chemistry and non-stoichiometry. NiOx is known to be a non-stoichiometric. The nickel, oxygen vacancies and the presence of Ni^3+^ can strongly influence the p-type carrier density. Moderate thermal treatment ~ 300 °C can increase the concentration of acceptor-like defects. For example, Ni vacancies or Ni^3+^ centres that supply holes, explaining the peak in carrier concentration^35^. At higher temperature > 400 °C, may reduce some defect types by promoting defect annihilation or cause grain coarsening that reduces the density of electrically active boundaries. This phenomenon leading to a lower apparent free carrier density. These behaviours are consistent with previous reports that relate the calcination temperature with non-stoichiometry and p-type conductivity^36^.

Furthermore, increasing calcination temperature generally improves crystallinity and reduces certain kinds of point defects, which can increase mobility by reducing trap-assisted scattering. However, excessive calcination can lead to grain growth and densification where can be observed at 400 °C NiOx sample, which reduces grain-boundary area that can sometimes contribute carriers via defect states. Large grains can also increase long-range scattering from structural inhomogeneities or create microcracks, reducing film continuity and effective mobility. Thus, an intermediate temperature ~ 300 °C can produce an optimal balance where sufficient crystallinity is needed to reduce trap density but retention of a favorable defect population that supplies carriers. This balance explains why $\:n$ peaks at 300 °C while µ does not necessarily increase in the same proportion.

## Conclusions

In this study, NiOx thin films were successfully synthesized via a surfactant-free, water-based co-precipitation method and calcinated at different temperatures to investigate the influence of thermal treatment on their structural, morphological, and optical properties. The FTIR analysis results indicate the production of NiOx from Ni(OH) entirely at 300 °C. XRD analysis revealed that at 300 °C, NiOx displayed a relatively small particle size (9.19 nm) with a homogeneous distribution compared to others. FESEM-EDX investigation demonstrates that the synthesized NiOx exhibits diverse morphological structures, contingent upon the calcination temperature. While some material behaviour observed here aligns with previous reports, the strength of this work lies in its practical focus, which is demonstrating a surfactant-free, water-based approach to synthesize and process NiOx with tuneable properties suitable for scalable photovoltaic applications. This study offers a useful framework for transitioning from nanoparticle synthesis to functional thin films in a reproducible and low-cost manner.

The synthesis of NiOx thin film was conducted using spin-coating NiOx on FTO glass. The structural, optical, and electrical properties of the thin film have been examined, revealing a uniform distribution of the FTO glass as observed in cross-sectional FESEM images. A cubic phase structure with a band gap of 3.81 eV and Urbach energy of 0.35 eV was identified using a UV-VIS study. The Hall effect analysis shows carrier concentration is the highest for NiOx prepared at 300 °C. It is well known that NiO can exhibit non-stoichiometric behaviour, and the presence of Ni³⁺ ions is often associated with darker coloration and enhanced p-type conductivity. In this study, the observed transition to black coloration at higher calcination temperatures suggests the possible formation of Ni³⁺ species. However, due to the absence of XPS analysis, the oxidation state of nickel in our samples cannot be definitively confirmed. Future work will include XPS characterization to further explore the Ni²⁺/Ni³⁺ ratio and its correlation with the electrical and optical properties of the NiOx thin films. While this study focused on the physicochemical optimization of NiOx films, future work will involve integrating these materials into perovskite solar cells to validate their effectiveness as hole transport layers and assess their impact on device efficiency and stability.

## Data Availability

The data that support the findings of this study are available from Mohammad Nur-E-Alam, upon reasonable request.
